# Implementation of convolutional neural networks for microbial colony recognition

**DOI:** 10.1128/spectrum.02885-24

**Published:** 2025-07-23

**Authors:** Fanhui Kong, Mingkuan Su, Jianfeng Guo, Haiying Wu, Hongbin Chen, Jie Qiu, Jiancheng Huang

**Affiliations:** 1 Department of Laboratory Medicine, Mindong Hospital of Ningde City 510255 , Ningde, Fujian, China; 2 Department of Laboratory Medicine, Mindong Hospital Affiliated to Fujian Medical University, Fuan, China; Emory University, Atlanta, Georgia, USA

**Keywords:** algorithm, colony, convolutional neural network, deep learning, microorganism

## Abstract

Initial classification of microorganisms based on visual identification of colonies remains challenging for skilled microbiologists and is influenced by the proficiency and subjective interpretation of professionals. To overcome these challenges, we applied deep learning to microbial colony recognition to provide microbial data to microbiologists to assist in clinical classification. Photographs of clinically isolated microbial colonies were captured to produce a 48 × 48 pixel colony data set, which was divided into training, validation, and test data sets. Eight convolutional neural networks (CNNs) were adapted to the colony classification task. The classification performance of the models was evaluated based on accuracy, precision, recall, and F1 score. The data set included five categories, namely gram-negative bacilli, gram-positive cocci, *Candida*, *Aspergillus*, and background of blood agar medium*,* with corresponding labels of 0, 1, 2, 3, and 4, respectively; each category contained 1,000 images. Among the trained CNNs, AlexNet showed the lowest performance, with an accuracy of 93.40%, whereas GoogLeNet had the highest performance, with an accuracy of 98.80%. MobileNet and ShuffleNet were more than 98% accurate. GoogLeNet misclassified only 6 of the 500 images in the test data set, and the algorithm was extremely capable of identifying both clinical and standard strains that were not included in the data set. The various trained CNNs demonstrated excellent performance in microbial colony recognition. These data-driven CNNs are expected to provide auxiliary decision-making tools for microbiologists.

**IMPORTANCE:**

Currently, the classification of microorganisms is highly subjective because it is dependent upon the skill level of the microbiologist. In this study, we used deep learning for microbial colony recognition to provide objective information to guide the identification of microbial colonies. We used photographs of clinically isolated microbial colonies divided into training, validation, and test data sets. Eight convolutional neural networks (CNNs) were applied, and the classification performance of each model was evaluated using accuracy, precision, recall, and F1 scores. Our study confirmed that CNNs can classify colonies into four broad categories: gram-negative bacilli, gram-positive cocci, *Candida*, and *Aspergillus*, with excellent predictive performance. Our method does not require specialized photographic equipment and exhibits high generalization performance, even for unknown bacteria.

## INTRODUCTION 

Although the names of microorganisms often change with advancing knowledge, numerous pathogens encountered in modern times have been recognized for centuries. Over the past few decades, molecular biology techniques (from polymerase chain reaction to next-generation sequencing) and mass spectrometry have emerged as key tools for pathogen identification in clinical microbiology laboratories (1, 2). However, routine microbial culture continues to be widely utilized in these laboratories as the primary method for isolating pathogens because of its effectiveness in recovering viable organisms and the availability of a given organism for antimicrobial susceptibility testing (3). A conventional microbial culture process involves propagation of pathogens under artificially created culture conditions on solid media and the formation of colonies that are visible to the naked eye (4). Microbiologists use colony characteristics or gram staining as methods to determine the gram-negative or gram-positive nature of a colony in order to facilitate subsequent experiments. Initial classification of microorganisms by visual identification of colony characteristics necessitates microbiologists to possess extensive practical experience, and the reliability of data is affected by the skill level of the evaluator (5). The Gram staining process comprises slide preparation, fixation, dyeing, and drying, and interpretation of the results remains labor- and time-intensive and highly operator-dependent (6). Large-scale data processing using these methods poses challenges.

Convolutional neural networks (CNN) have been applied in the medical field; CNN can automatically learn complex feature representations using images and realize high-precision classification and detection in various image-recognition tasks (7). A deep learning study published in 2016 trained a diabetic retinopathy diagnostic model using 128,175 retinal images to perform diagnoses, with the results comparable to those determined by ophthalmologists (8).

With the emergence of digital microbiology, artificial intelligence (AI) has been integrated into clinical microbiology practice (9). Deep learning has been applied in urine culture image analysis (10) and bacterial colony counting (11) and has shown excellent performance. However, the use of deep learning for identifying microbial colonies remains limited. Therefore, the aim of this study was to establish CNNs for colony identification to enhance the utilization of microbial data.

## RESULTS

### Microbial composition

From January to June 2024, 11 genera of common pathogenic microorganisms were isolated from clinical samples. The 11 isolated genera were *Escherichia*, *Klebsiella*, *Pseudomonas*, *Acinetobacter*, *Stenotrophomonas*, *Enterobacter*, *Staphylococcus*, *Enterococcus*, *Streptococcus*, *Candida*, and *Aspergillus*. The Gram staining results for these microorganisms are shown in Table 1.

**TABLE 1:** Basic information from digital images of the microbial colony data set

Label	Microorganism	Gram stain	Original image	Data set image, *n* (%)
0	Gram-negative bacilli	Negative	124	1,000 (20.0)
	*Escherichia*	Negative	39	300 (6.0)
	*Klebsiella*	Negative	20	200 (4.0)
	*Pseudomonas*	Negative	25	200 (4.0)
	*Acinetobacter*	Negative	14	100 (2.0)
	*Stenotrophomonas*	Negative	15	100 (2.0)
	*Enterobacter*	Negative	11	100 (2.0)
1	Gram-positive cocci	Positive	116	1,000 (20.0)
	*Staphylococcus*	Positive	53	400 (8.0)
	*Enterococcus*	Positive	41	300 (6.0)
	*Streptococcus*	Positive	22	300 (6.0)
2	*Candida*	Positive	82	1,000 (20.0)
3	*Aspergillus*	Positive	71	1,000 (20.0)
4	Background	/	/^*a*^	1,000 (20.0)

^*a*^ Randomly selected 100 images from 393 original images as the background image sources.

### Digital images of microbial colony

**Fig 1:**
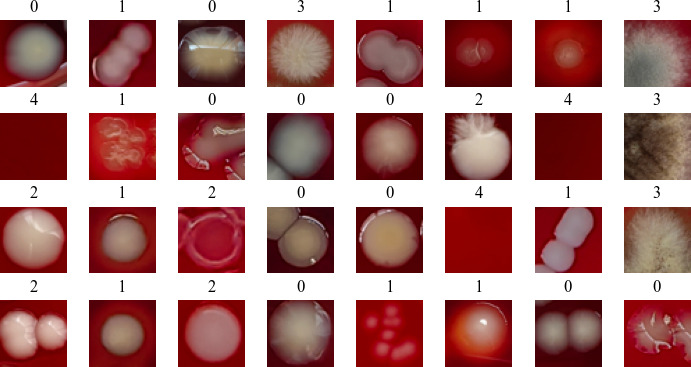
Batch of images randomly obtained digital images from the microbial colony data set. Annotations: 0, gram-negative bacillus; 1, gram-positive coccus; 2, *Candida*; 3, *Aspergillus*; 4, background of blood agar medium.

A total of 393 original images (6,144 × 8,192 pixels) were captured, and each original image was unique. We obtained 5,000 images with a resolution of 48 × 48 pixels by cropping 1,000 images for each label category. Individual strains were cropped to no more than 20 images to reduce the proportion of images of each strain in the data set. The data set was divided into training, validation, and test data sets at a ratio of 8:1:1, and the images in each sub-data set were unique. The data set included five categories: gram-negative bacillus, gram-positive coccus, *Candida*, *Aspergillus*, and background of blood agar medium, with corresponding labels of 0, 1, 2, 3, and 4, respectively. The microbial species and digital image composition ratios of each label category are shown in Table 1. The data set produced was designated as the digital image of the microbial colony (DIMC) data set. Figure 1 shows a randomly acquired batch of 32 images and demonstrates that the 48 × 48 pixel images included colonies that ranged from one colony to tens of colonies or fused colonies in patches.

### CNN training and performance evaluation

**Fig 2:**
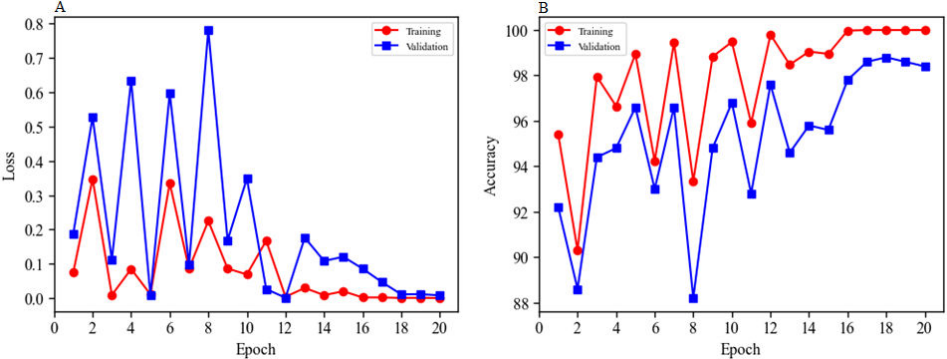
Loss and accuracy line plots for GoogLeNet training. Annotation: (**A**) line plot of loss and number of epochs; (**B**) line plot of accuracy and number of epochs.

PyTorch (https://pytorch.org/) provides powerful data-loading and preprocessing capabilities that make it easy for users to load and preprocess data. To begin training the CNN, the DIMC data set was loaded, and the image was standardized, normalized, and converted to a tensor. To reduce memory consumption and accelerate training, batch training was performed with 32 images in each batch. The parameters were saved at the end of CNN training, and line plots were used to visualize accuracy and loss during the training process. The training process for GoogLeNet is shown in Fig. 2. Figure 2A shows that after 17 iterations of the model during the training process, loss decreased rapidly in both the training and validation data sets and gradually converged to a very small interval, whereas its accuracy increased gradually for both the training and validation data sets and eventually converged to a very small interval (Fig. 2B). To assess the generalizability of the proposed algorithm, a test data set was used to verify its performance.

**Fig 3:**
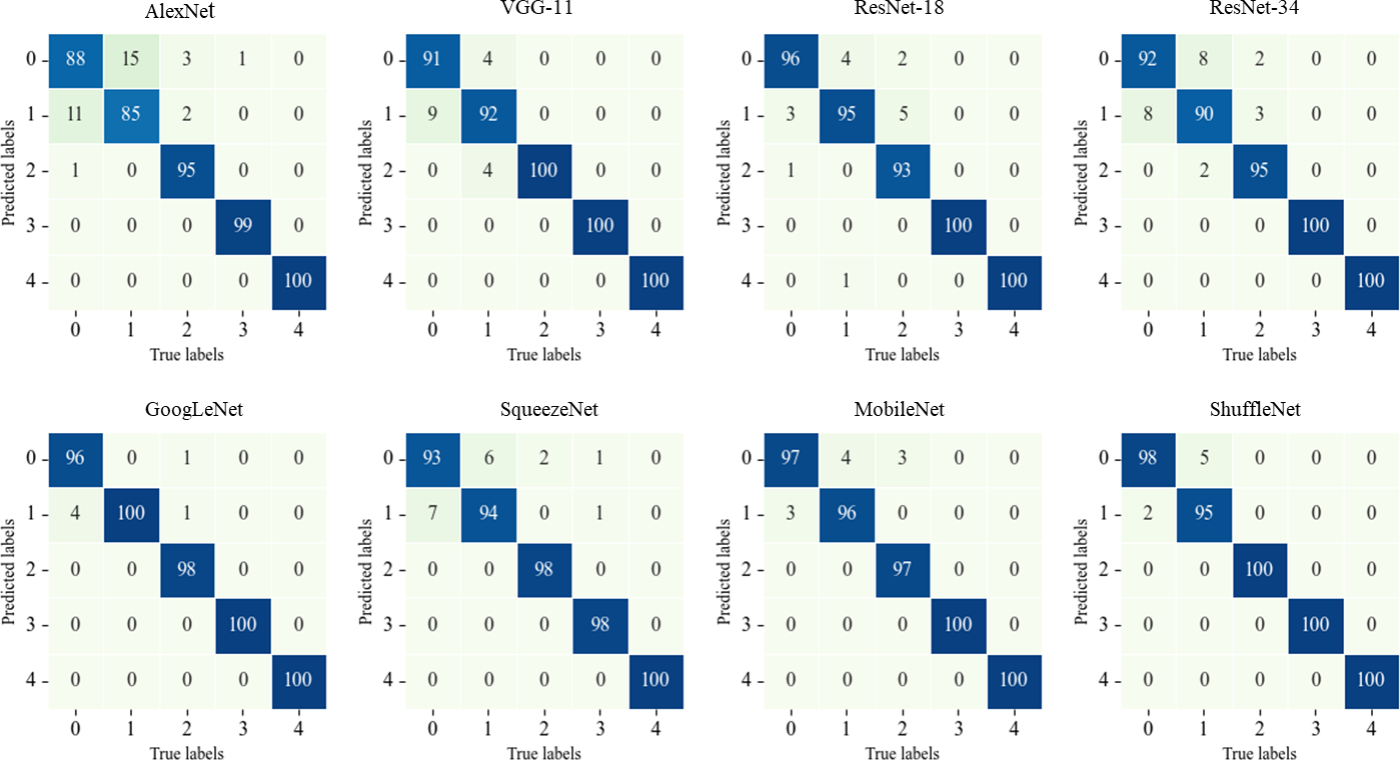
Confusion matrix of convolutional neural network prediction results for the test data set.

Table 2 lists the prediction results of the eight CNNs in the test data set. GoogLeNet exhibited the best performance with an accuracy of 98.80%. ShuffleNet and MobileNet ranked second and third, with accuracies of 98.60% and 98.00%, respectively. ResNet-18 had an accuracy of 96.80%, and ResNet-34 had a deeper network structure than ResNet-18, whereas its accuracy was 95.40%. VGG-11 and AlexNet showed accuracies of 96.80% and 93.40%, respectively. Because of the performance of the graphics processing unit (GPU), the GeForce GTX 1650 (NVIDIA, Santa Clara, CA, USA) was unable to run VGG-11. The training time to run VGG-11 with the CPU was 46.75 minutes, whereas that for the remaining seven CNNs via the GPU was a maximum of 14 minutes. ShuffleNet and GoogLeNet, which exhibited excellent predictive performance, required a training time of less than 10 minutes. The rows of the confusion matrix represent the true labels, and the columns represent the predicted labels. Figure 3 lists the confusion matrices of the eight CNNs. GoogLeNet had six image classification errors for the test data set and showed 100% accuracy in identifying gram-positive cocci, *Aspergillus*, and background.

**TABLE 2:** Training results and performance comparison of convolutional neural networks

Model	Test data set				Training accuracy (%)	Validation accuracy (%)	Test accuracy (%)	Training time (min)
	Label	Precision	Recall	F1 score				
AlexNet	0	0.82	0.88	0.85	97.95	91.60	93.40	13.64
	1	0.87	0.85	0.86				
	2	0.99	0.95	0.97				
	3	1.00	0.99	0.99				
	4	1.00	1.00	1.00				
VGG-11	0	0.96	0.91	0.93	98.52	96.60	96.60	46.75
	1	0.91	0.92	0.92				
	2	0.96	1.00	0.98				
	3	1.00	1.00	1.00				
	4	1.00	1.00	1.00				
ResNet-18	0	0.94	0.96	0.95	100.00	96.60	96.80	10.04
	1	0.92	0.95	0.94				
	2	0.99	0.93	0.96				
	3	1.00	1.00	1.00				
	4	0.99	1.00	1.00				
ResNet-34	0	0.90	0.92	0.91	99.83	95.20	95.40	11.44
	1	0.89	0.90	0.90				
	2	0.98	0.95	0.96				
	3	1.00	1.00	1.00				
	4	1.00	1.00	1.00				
GoogLeNet	0	0.99	0.96	0.97	100.00	98.40	98.80	9.02
	1	0.95	1.00	0.98				
	2	1.00	0.98	0.99				
	3	1.00	1.00	1.00				
	4	1.00	1.00	1.00				
SqueezeNet	0	0.91	0.93	0.92	99.97	95.80	96.60	8.52
	1	0.92	0.94	0.93				
	2	1.00	0.98	0.99				
	3	1.00	0.98	0.99				
	4	1.00	1.00	1.00				
MobileNet	0	0.93	0.97	0.95	100.00	96.80	98.00	9.58
	1	0.97	0.96	0.96				
	2	1.00	0.97	0.98				
	3	1.00	1.00	1.00				
	4	1.00	1.00	1.00				
ShuffleNet	0	0.95	0.98	0.97	100.00	99.00	98.60	9.31
	1	0.98	0.95	0.96				
	2	1.00	1.00	1.00				
	3	1.00	1.00	1.00				
	4	1.00	1.00	1.00				

### Prediction of standard strains and clinically isolated strains not included in the data set by GoogLeNet

The DIMC data set did not include the standard strains *Escherichia coli* (*E. coli*), *Klebsiella pneumonia* (*K. pneumonia*), *Pseudomonas aeruginosa* (*P. aeruginosa*), *Staphylococcus aureus* (*S. aureus*), and *Streptococcus pneumoniae* (*S. pneumoniae*). GoogLeNet accurately predicted all the above standard strains as gram-negative bacilli (*E. coli*, *K. pneumonia*, and *P. aeruginosa*) or gram-positive cocci (*S. aureus* and *S. pneumoniae*).

*Serratia marcescens*, *Klebsiella oxytoca*, *Citrobacter braakii*, *Burkholderia cepacia*, *Morganella morganii*, *Raoultella ornithinolytica*, *Vibrio parahaemolyticus*, *Enterococcus avium*, *Enterococcus casseliflavus*, *Enterococcus hirae*, *Staphylococcus haemolyticus*, *Streptococcus viridans*, *Streptococcus dysgalactiae*, *Streptococcus sanguinis*, *Streptococcus gordonii*, *Candida lusitaniae*, *Candida lipolytica*, and *Candida parapsilosis* were not included in the DIMC data set. These clinical isolates were used to further evaluate the ability of GoogLeNet to identify unknown strains.

The above standard strains and clinical isolates were designated as the second test data set, with a total of 230 images, including 100 images of gram-negative bacilli, 100 images of gram-positive cocci, and 30 images of *Candida* (10 images for each strain) (Table 3). Using GoogLeNet for the second test data set, two images of gram-negative bacilli were predicted as gram-positive cocci, three images of gram-positive cocci were predicted as gram-negative bacilli, and one image of gram-positive cocci was predicted as background, with a prediction accuracy of 97.39%, indicating that GoogLeNet can accurately recognize unknown bacteria. The images and predicted probabilities of the second test data set are presented in Fig. S1 and Table S1.

**TABLE 3:** Basic information for images in the second test data set

Image name	Microbial name	Gram stain	True label
Figure S001–010	*Escherichia coli* (ATCC25922)	Negative	0
Figure S011–020	*Klebsiella pneumoniae* (ATCC13883)	Negative	0
Figure S021–030	*Pseudomonas aeruginosa* (ATCC27853)	Negative	0
Figure S031–040	*Serratia marcescens*	Negative	0
Figure S041–050	*Klebsiella oxytoca*	Negative	0
Figure S051–060	*Citrobacter braakii*	Negative	0
Figure S061–070	*Burkholderia cepacia*	Negative	0
Figure S071–080	*Morganella morganii*	Negative	0
Figure S081–090	*Raoultella ornithinolytica*	Negative	0
Figure S091–100	*Vibrio parahaemolyticus*	Negative	0
Figure S101–110	*Staphylococcus aureus* (ATCC25923)	Positive	1
Figure S111–120	*Streptococcus pneumoniae* (ATCC6305)	Positive	1
Figure S121–130	*Enterococcus avium*	Positive	1
Figure S131–140	*Enterococcus casseliflavus*	Positive	1
Figure S141–150	*Enterococcus hirae*	Positive	1
Figure S151–160	*Staphylococcus haemolyticus*	Positive	1
Figure S161–170	*Streptococcus viridans*	Positive	1
Figure S171–180	*Streptococcus dysgalactiae*	Positive	1
Figure S181–190	*Streptococcus sanguinis*	Positive	1
Figure S191–200	*Streptococcus gordonii‌*	Positive	1
Figure S201–210	*Candida lusitaniae*	Positive	2
Figure S211–220	*Candida lipolytica*	Positive	2
Figure S221–230	*Candida parapsilosis*	Positive	2

## DISCUSSION

In 2012, Krizhevsky et al. (12) proposed a deep neural network called AlexNet. This algorithm won the ImageNet large-scale visual recognition challenge with a clear margin. The success of AlexNet has facilitated the application of deep learning in various fields, such as image recognition and natural language processing. In subsequent years, deep learning methods such as VGGNet, GoogLeNet, and ResNet set the best records in the ImageNet competition (13). In computer vision, transferred learning enables CNNs trained on large-scale data sets to migrate to specific types of image-recognition tasks (14). We fine-tuned eight pre-trained CNNs for microbial colony recognition using transferred learning. Our results showed that GoogLeNet exhibited an accuracy of 98.80%; only 6 images had a classification error for a test data set of 500 images, compared to the 93.40% accuracy of classical AlexNet. Although VGG-11 is a shallower CNN in the VGGNet family, it exhibits a more complex network structure than AlexNet. This increased complexity results in a larger number of layers and parameters, necessitating more computational resources and a longer training time for VGG-11. Our results showed that the performance of ResNet-34 was marginally lower than that of ResNet-18, indicating that increasing the network depth does not improve the accuracy of microbial colony identification. GoogLeNet adopts a 22-layer deep network structure, and its parameters of 50 Mb have been significantly reduced compared to those of AlexNet and VGGNet, which can be up to hundreds of Mb. GoogLeNet achieved the highest accuracy in the test data set. MobileNet, ShuffleNet, and SqueezeNet are lightweight deep neural networks with fewer parameters and faster training speeds while maintaining relatively high accuracy. The number of parameters in the ShuffleNet model was 8.79 Mb, which was considerably lower than that of GoogLeNet; however, its accuracy was similar to that of GoogLeNet. Constrained by the performance of the GPU, we were unable to train deep neural networks with very large parameters; however, the above lightweight deep neural networks provide an effective solution when using resource-constrained devices. For example, Zhang et al. (15) showed that the YOLOv3 network can be deployed to edge computing devices and uses smartphone cameras to achieve on-site testing of colony counts, greatly reducing the cost of data collection and annotation.

Microbial colonies form through interactions between their genotypes, phenotypes, and environments and are characterized by their size, shape, edges, texture, transparency, and color. The superior performance of deep learning in the classification of various image data sets has enabled microbial colony classification. Zieliński et al. (16) demonstrated that texture recognition can effectively classify bacterial genera and species by applying Gram stain to colonies and classifying the acquired cell images. The method of obtaining images by pure colony smear Gram staining resulted in images that were too “clean” and did not consider the presence of human cells in primary specimens (e.g., sputum) or the interference from other components. These limitations may not be generalizable to the identification of bacterial cells through smear Gram staining of the primary specimens. An additional step in Gram staining is polymerization, particularly when it is utilized to classify colonies after culture. Rattray et al. (17) used microphotography technology to photograph 69 strains of *P. aeruginosa* colonies from environmental and clinical isolates to determine the microscale characteristics of the colonies and showed that the average accuracy of classification of individual strains from both the validation and test data sets exceeded 90%. The authors suggested that bacterial strains have characteristic visual “fingerprints” that can be used as a basis for classification at the subspecies level. However, the study required a high-resolution imaging system and a complex process for processing the raw images, and the study was not extended to other bacteria; therefore, there are no data to support the existence of similar visual “fingerprints” for *P. aeruginosa* in other bacteria.

Our fine-tuned CNNs are pre-trained on the ImageNet-1000 data set, which contains 1.4 million images and 1,000 categories, each with 1,000 images. Our data set has five categories, and the number of images in each category is equivalent to that of the ImageNet-1000 data set. Our study confirmed that CNNs can classify colonies into four broad categories of gram-negative bacillus, gram-positive coccus, *Candida*, and *Aspergillus*, with excellent predictive performance. Additionally, the images will not be mistaken for the background of the blood agar medium, indicating the ability of CNNs to accurately identify colonies.

However, colony morphology is one colony characteristic, and catalase and oxidase tests are also classic methods for rapid biochemical identification of microorganisms. The catalase test is capable of differentiating between *Staphylococcus* and *Streptococcus*, and the oxidase test is mainly used to identify *Pseudomonas* and Enterobacteriales. The catalase and oxidase tests are only applicable if the colony is determined to be gram-positive or gram-negative. Rapid biochemical identification methods and deep learning are complementary rather than substitute tests. If the deep learning model determines that the colony is gram-negative bacillus and the oxidase test is positive, the colony can be preliminarily identified as *Pseudomonas*. If the deep learning model determines that the colony is gram-positive coccus, it can be further determined that the colony is *Staphylococcus* or *Streptococcus* using the catalase test. Our research supports the idea that AI applications can increase productivity and improve processes in clinical microbiology laboratories. Our study did not require specialized photographic equipment and showed a very high generalization performance, even for unknown bacteria. In addition, if it is necessary to expand the data set to achieve more categories of microbial classification, only the output layer of the CNNs must be modified, and no other parameter modifications are involved. Some scholars are currently applying AI platforms, such as the WASPLab platform (18, 19) and APAS Independence (5, 20), which can be used for the interpretation of urine culture and the detection of methicillin-resistant *S. aureus* on chromogenic medium with a sensitivity of 100%. In the future, microbial laboratories will increasingly utilize a combination of AI and microbial automation workstations for enhanced efficiency and standardization.

Our study had some limitations. Given the performance of GPUs, we were unable to train deep neural networks with an extensive number of parameters; the CNNs lacked validation with external data, and the data set incorporated clinically common microorganisms without sufficient diversity.

In summary, we used CNNs to identify microbial colonies and found that multiple algorithms achieved excellent performance. Our study provides reliable data for microbial digitization. Future work will focus on expanding the data set to achieve more microbial colony classification categories.

## MATERIALS AND METHODS

### Strain source and identification

Specimens collected in the clinical microbiology laboratory were inoculated onto a blood agar plate. The plates were incubated in 5% CO_2_ at 35℃ for 24–48 hours according to the purpose of the culture. Strains isolated by routine culture, as described above, were identified using a VITEK 2 (bioMérieux, Marcy-l’Étoile, France), and *Aspergillus* was identified using the lactophenol cotton blue staining method. Quality control strains included *E. coli* (ATCC 25922; Manassas, VA, USA), *K. pneumoniae* (ATCC 13883), *P. aeruginosa* (ATCC 27853), *S. pneumoniae* (ATCC 6305), and *S. aureus* (ATCC 25923).

### Computer hardware and software configuration

An Intel Core 6-core i5-8400 @ 2.80 GHz platform (Intel, Santa Clara, CA, USA) was used with the Windows 10 operating system (Microsoft, Redmond, WA, USA). A NVIDIA GeForce GTX 1650 graphics card with CUDA 12.1 was used. The programming language used was Python 3.11.7 (https://www.python.org/), the deep learning framework was PyTorch (https://pytorch.org/), and the editor was Jupyter Notebook (https://jupyter.org/).

### Digital image acquisition and data preprocessing

The blood agar plate was placed on a white background and photographed using a Huawei Mate 50 camera (high-pixel mode) (Huawei, Guangdong, China) at a height of 12 cm, and only one original image was taken. The original image was captured at a resolution of 6,144 × 8,192 pixels and had a size of 10 Mb. The image was opened using a photo reader and resized to a suitable size (10%–20% of the original image), and the target area was cropped using the screen capture tool FSCapture (FastStone Software Technologies, Inc., Essex, UK).

### Deep learning via transferred learning and performance assessment

PyTorch is an open-source machine learning library that supports NVIDIA CUDA, enabling it to perform efficient computations on a GPU and speed up the training process. Taking advantage of transferred learning and architectural diversity, we loaded eight CNNs from PyTorch, which included AlexNet, VGG-11, ResNet-18, ResNet-34, SqueezeNet, GoogLeNet, MobileNet V_2_, and ShuffleNet V_2_. All CNNs were pre-trained on the ImageNet data set, and their parameter capacities were 233, 506, 44.6, 83.2, 4.72, 49.7, 13.5, and 8.79 Mb, respectively. The output layers of the CNNs contained 1000 neurons and were modified to five neurons to fit the microbial colony recognition task. The classification performances of the above CNNs were evaluated based on accuracy, precision, recall, and F1 score.

## Data Availability

The raw data are available on reasonable request from the corresponding author.

## SUPPLEMENTAL MATERIAL

Figure S1 spectrum.02885-24-s0001.tif Colony images of standard strains and clinical isolates.

Supplemental legend spectrum.02885-24-s0002.docx Legend for Fig. S1.

Table S1 spectrum.02885-24-s0003.xlsx Predicted probabilities of all images in the second test data set.

ASM does not own the copyrights to Supplemental Material that may be linked to, or accessed through, an article. The authors have granted ASM a non-exclusive, world-wide license to publish the Supplemental Material files. Please contact the corresponding author directly for reuse.
