# Supplementary figures and images for: Implementation of convolutional neural networks for microbial colony recognition
Source: Microbiol Spectr. 2025 Jul 23;13(9):e02885-24. doi: 10.1128/spectrum.02885-24 (PMC12403611; doi:10.1128/spectrum.02885-24)

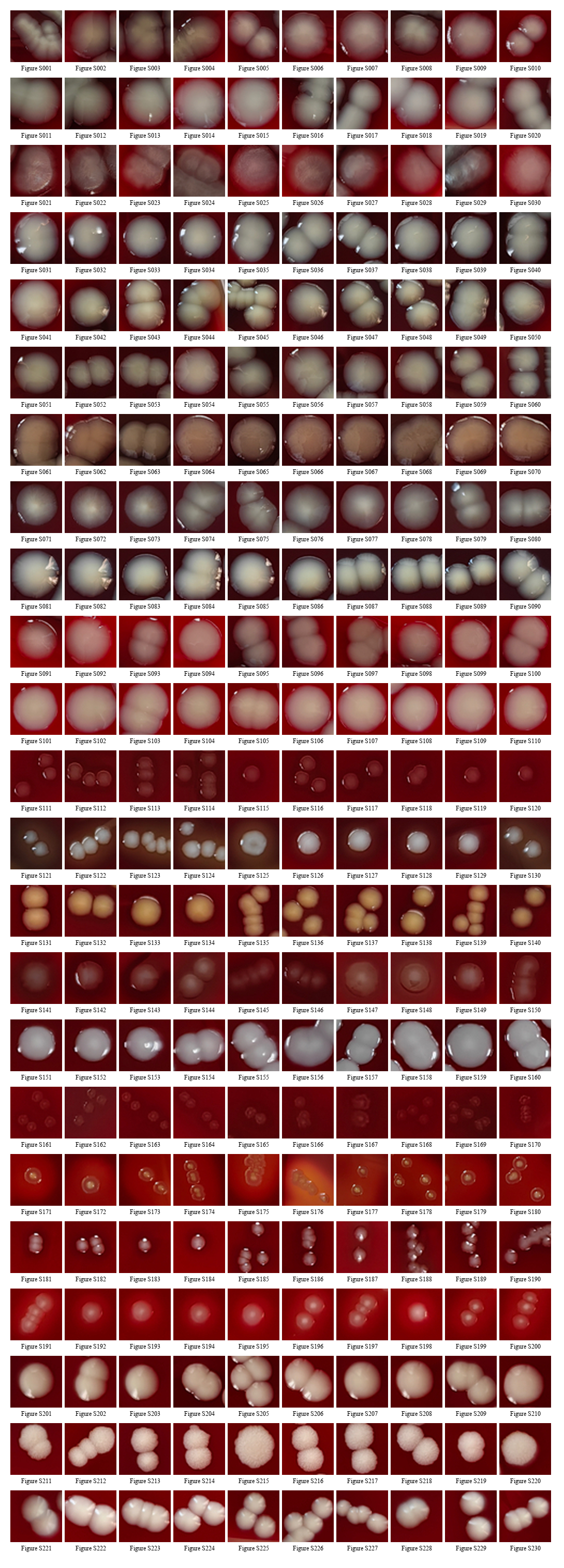

Supplement: Figure S1 — Colony images of standard strains and clinical isolates. [file spectrum.02885-24-s0001.tif]
