# Supplementary material for: Implementation of convolutional neural networks for microbial colony recognition
Source: Microbiol Spectr. 2025 Jul 23;13(9):e02885-24. doi: 10.1128/spectrum.02885-24 (PMC12403611; doi:10.1128/spectrum.02885-24)
Supplement: Supplemental legend — Legend for Fig. S1. [file spectrum.02885-24-s0002.docx]

**Supplementary figure captions**

**Figure S1:** Colony images of standard strains and clinical isolates. Annotation: Microbial names corresponding to Figure S001-230 are shown in Table 3.
